# Antimicrobial susceptibility and molecular characteristics of *Mycoplasma pneumoniae* isolates across different regions of China

**DOI:** 10.1186/s13756-019-0576-5

**Published:** 2019-08-23

**Authors:** Fei Zhao, Jing Li, Jinrong Liu, Xuemei Guan, Jie Gong, Liyong Liu, Lihua He, Fanliang Meng, Jianzhong Zhang

**Affiliations:** 1 National Institute for Communicable Disease Control and Prevention, Chinese Center for Disease Control and Prevention, State Key Laboratory of Infectious Disease Prevention and Control, 155 Changbai Road, Changping District, Beijing, 102206 China; 2 Office of Laboratory Management, Chinese Center for Disease Control and Prevention, Beijing, 102206 China; 3 Department of Respiratory Medicine, Beijing Children’s Hospital, National Center for Children’s Health, Capital Medical University, Nanlishi Road 56, Xicheng District, Beijing, China; 4 Affiliated Hospital of Beihua University, Jilin, 132011 China

**Keywords:** *Mycoplasma pneumoniae*, Macrolide resistance, 23S rRNA gene, Genotype

## Abstract

**Background:**

In China mainland, most *Mycoplasma pneumoniae* related studies are carried out in Beijing and Shanghai, while rare studies are performed in the other regions. In this study, we analyzed the molecular biology characteristics and antimicrobial susceptibility of clinical isolates of *M. pneumoniae* from 5 regions between January 2017 and December 2018.

**Methods:**

Genotyping was performed to 154 *M. pneumoniae* isolates from 5 cities using PCR and multiple-locus variable-number tandem repeat analysis (MLVA) method. Antimicrobial susceptibility test was performed to all the isolates against 4 antibiotics. Sequencing was performed to the amplification products of the 23S rRNA drug resistant gene.

**Results:**

Genotype I was detected in 118 *M. pneumoniae* isolates (76.6%), and genotype II was identified in 36 isolates (23.4%). The majority (92.2%) of the MLVA genotypes were 4–5–7-2 and 3–5–6-2, which represented the genotype I and II, respectively. The total macrolide (ML) resistance rate was 79.7%. The minimum inhibitory concentration (MIC) of the erythromycin was in a range of 128- > 256 μg/ml, while that for the azithromycin was 2-32 μg/ml. There were mutations in the 23S rRNA in each ML resistance isolate. Jilin city showed the highest prevalence of genotype I (100%) and ML resistance rate (100%), while Jinan showed the lowest prevalence of genotype I (45.5%) and ML resistance rate (54.5%).

**Conclusions:**

A large variance was identified in the *M. pneumoniae* genotype and ML resistance among the 5 cities. The proportion of *M. pneumoniae* with a genotype II genotype (3–5–6-2) showed an increased trend.

**Electronic supplementary material:**

The online version of this article (10.1186/s13756-019-0576-5) contains supplementary material, which is available to authorized users.

## Background

*Mycoplasma pneumoniae*, an important pathogenic bacteria associated with respiratory tract infection in human, is responsible for about 10 to 40% of the community acquired pneumonia (CAP) [1, 2]. *M. pneumoniae* infection is usually considered as a self-limited disease, while a part of patients may develop severe pneumonia especially the children [3, 4].

Outbreak of *M. pneumoniae* infections also tends to occur in different regions with cyclic epidemics every 3–7 years. Since 2010, outbreak of *M. pneumoniae* has been reported in some European countries, including Denmark [5], Norway [6], Germany [7], United Kingdom and Wales [8], France [9] and Finland [6]. Afterwards, similar reports are available in Chile [10], Israel [11], Korea [12], Japan [13] and China [14]. Indeed, these studies reported the biological features of *M. pneumoniae* including population characteristics, genotype and antimicrobial susceptibility. Nevertheless, little is known about the relationship between *M. pneumoniae* epidemicity and genotype, antimicrobial susceptibility and virulence factors. This leads to urgent demands on the epidemiological and molecular analysis.

In China mainland, most of the *M. pneumoniae* related studies are conducted in Beijing and Shanghai [15–19], while extremely rare studies are performed in the other regions. In a recent study, Xue et al. [20] firstly reported the *M. pneumoniae* genotype and antimicrobial resistance in 6 regions simultaneously. However, the *M. pneumoniae* genotype and antimicrobial resistance were derived from *M. pneumoniae* DNA detection, not from the clinical isolates. In this study, we analyzed the genotype and antimicrobial susceptibility of clinical isolates of *M. pneumoniae* from 5 regions between January 2017 and December 2018.

## Material and methods

### M. pneumoniae strains

**Fig. 1:**
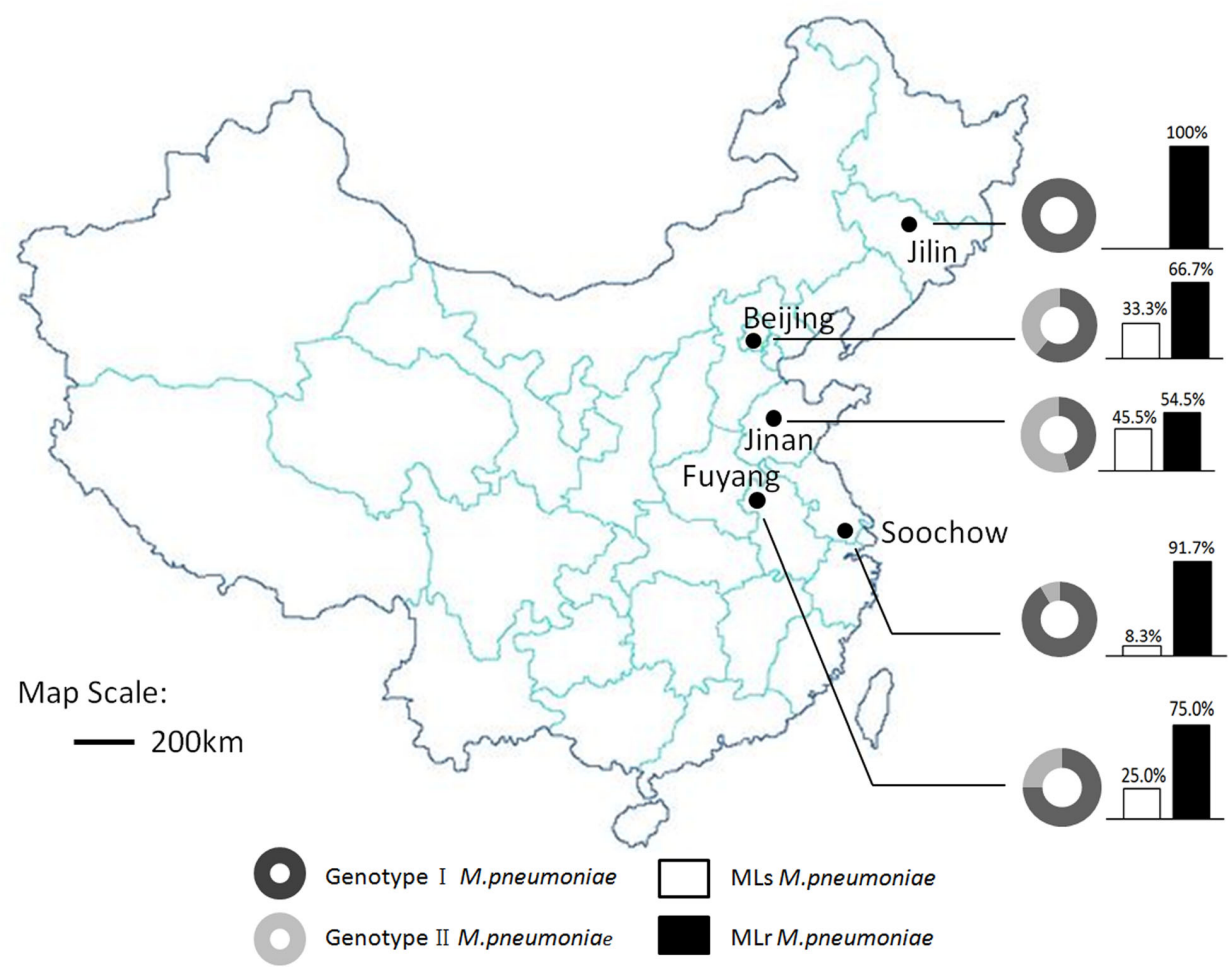
Locality map, genotype and ML resistant rate of five cities included in this study

*M. pneumoniae* strains were provided by the National Institute for Communicable Disease Control and Prevention, Chinese Center for Disease Control and Prevention. A total of 154 strains were collected from 267 pediatric patients preliminarily confirmed with *M. pneumoniae* pneumonia in local hospitals in 5 cities from north to south of China, including Jilin (Jilin Province, China), Beijing, Jinan (Shandong Province, China), Fuyang (Anhui Province, China) and Soochow (Jiangsu Province, China) **(**Fig. 1**)**. Each throat swab specimen was cultured in 2 ml *Mycoplasma* selective liquid media (Bioreal Coming) at 37 °C. In cases of media color changing from red to yellow, 0.1 ml suspension was transferred onto agar to subculture. Then *M. pneumoniae* isolates were purified using dilution technique. Nucleotide identification from each purified isolate was verified by Real-Time PCR as previously described [21].

### Real-time PCR-based genotyping

Genotyping of *M. pneumoniae* was performed by Real-Time PCR. Amplification of the specific regions of the genotype from all isolates was performed as previously described [22]. Real-time PCR was performed on the CFX96 system (Bio-Rad, Hercules, CA, USA). The amplification conditions were as follows: 95 °C for 2 min; followed by 45 cycles at 95 °C for 15 s and 56 °C for 15 s. The data were analyzed with the CFX Manager Software (version 3.1; Bio-Rad).

### Multiple-locus variable-number tandem repeat analysis (MLVA) genotyping

DNA was extracted from the 154 isolates using commercial QIAamp DNA MINI kit (QIAGEN, No. 51306), according to the manufacturer’s instructions. The DNA was utilized as the template for Multiplex PCR amplification-linked capillary electrophoresis of four loci (i.e. Mpn13–16) selected for multilocus variable-number tandem-repeat (VNTR) analysis, according to the previous description with moderate modifications [23].

### Antimicrobial susceptibility testing

Minimum inhibitory concentrations (MICs) against 4 antibiotics (i.e. erythromycin, azithromycin, levofloxacin and tetracycline) (Sigma-Aldrich, CA, USA) were determined using SP4 broth (Remel) based on the micro-dilution methods. *M. pneumoniae* M129 (ATCC 29342) and ICDC P028 (clinical isolate) served as macrolide-susceptible and -resistant controls, respectively. MIC was defined as the lowest concentration of antimicrobial agents that induced a color change in the control media. The MIC test was conducted based on the latest version of CSLI M43-A (2017 version). Sterilized liquid paraffin oil was utilized to seal the micro-plates, and then was incubated at 37 °C for 5 days. All the susceptibility tests were performed at least in triplicate.

### Amplification and sequencing domains V of the 23S rRNA gene

Domain V of the 23S rRNA gene was amplified using the primers according to the previous study [24]. Each amplification was performed in a total volume of 20 μl, containing 2 μl 10× Ex Taq Buffer (Mg^2+^ plus), 0.4 μM primers, 1.6 μl dNTP mixture, 0.2 μl TaKaRa Ex Taq (5 U/μl), and 1 μl of DNA template. Amplification was performed using the following conditions: 95 °C for 3 min; followed by 30 cycles of 95 °C for 30 s, 55 °C for 30 s, and 72 °C for 60 s; and 72 °C for 5 min. After purification, the obtained products were sequenced using Sanger technique (Sangon Biotech, Beijing, China).

### Statistical analysis

The data were entered into Excel 2007 sheet. Then SPSS 17.0 software was utilized for the statistical analysis. Chi square test was performed for the analysis of genotyping and drug resistance of strains isolated from different regions. *P* value of less than 0.05 was considered to be statistically significant.

## Results

### Real-time PCR genotyping

Genotype I was detected in 118 *M. pneumoniae* isolates (76.6%), and genotype II was identified in 36 isolates (23.4%, Table 1). The proportion of genotype I and II showed differences across various cities. The proportion of genotype I and II in Beijing was 60.8% (31/51) and 39.2%, respectively. The proportion of genotype I and II in Soochow was 91.7% (22/24) and 8.3% (2/24), respectively. In Fuyang, the proportions for genotype I and II were 75% (6/8) and 25% (2/8), respectively. In Jinan, the proportions were 45.4% (10/22) and 54.5% (12/22), respectively. In Jilin, the proportion for genotype I was 100% (49/49), and no genotype II was identified. Statistical analysis indicated that there were significant differences in the genotype of the *M. pneumoniae* in different regions (χ^2^ = 37.068, *P* < 0.05). Taken together, the distribution of the two genotypes was different in these cities **(**Fig. 1**)**.

**Table 1:** Molecular characteristics of Mycoplasma pneumoniae from five areas in China

	Total	Beijing	Jilin, JiLin	Soochow, Jiangsu	Jinan, Shangdong	Fuyang, Anhui
*M. pneumoniae* isolates	154	51	49	24	22	8
PCR genotypes						
Genotype I	118	31	49	22	10	6
Genotype II	36	20	0	2	12	2
MLVA genotypes						
4–5–7-2	107	29	43	20	10	5
4–5–7-3	7	1	4	2	0	0
4–4–7-2	4	1	2	0	0	1
3–5–6-2	35	20	0	2	11	2
3–6–6-2	1	0	0	0	1	0
Macrolide						
Resistance	123	34	49	22	12	6
Sensitive	31	17	0	2	10	2

### MLVA genotyping

The most common MLVA genotype was 4–5–7-2 (69.4%, 107/154), followed by genotype 3–5–6-2 (22.7%, 35/154), 4–5–7-3 (4.5%, 7/154), 4–4–7-2 (2.6%, 4/154), and 3–6–6-2 (0.6%, 1/154). Table 1 showed the MLVA genotyping of isolated obtained in each city. In terms of the traditional genotypes (i.e. genotype I and II), compared with the MLVA genotypes, more than 90% of the genotype I *M. pneumoniae* isolates belonged to 4–5–7-2, and genotype II isolates belonged to 3–5–6-2, respectively (Table 2).

**Table 2:** Relationship between traditional genotype and MLVA genotype of *M. pneumoniae*

Traditional genotype	MLVA genotype		
	Genotype	Numbers	%
Genoype I (118)	4–5–7-2	107	90.7
	4–5–7-3	7	5.9
	4–4–7-2	4	3.4
Genotype II (36)	3–5–6-2	35	97.2
	3–6–6-2	1	2.8

### Antimicrobial susceptibility testing of *M. pneumoniae*

In total, 123 (79.9%) were macrolide-resistant. The isolates collected in Jilin showed the highest macrolide-resistance rate (100%), followed by Soochow (91.7%), Fuyang (75%), Beijing (66.7%), and Jinan (54.5%, Table 1**,** Fig. 1). There were significant differences in the ML resistance of *M. pneumoniae* in different regions (χ^2^ = 28.851, *P* < 0.05). The 123 macrolide-resistant isolates and the reference strain ICDCP028 were resistant to erythromycin (MIC ≥128 μg/ml) and azithromycin (MIC 2–32 μg/ml). The other 31 isolates and reference strain M129 were susceptible to macrolide with a MIC of ≤0.008 for erythromycin and azithromycin. Each isolate was susceptible to tetracycline and levofloxacin (Table 3) (Additional file 1).

**Table 3:** Genotype characteristics and MIC ranges of four antimicrobial agents against 154 *M. pneumoniae* clinical isolates from five areas, 2017 to 2018

Region	Mutation in the 23S rRNA	Isolates number		MIC (μg/ml)			
		Genotype I	Genotype II	ERY	AZM	LVX	TET
Beijing	A2063G	30	4	128- > 256	2–32	0.125–1	0.016–0.125
	None	1	16	< 0.008	< 0.008	0.125–0.5	0.032–0.125
Jilin, Jilin	A2063G	48	0	128- > 256	2–32	0.125–0.5	0.016–0.25
	A2064G	1	0	> 256	2	0.5	0.125
Soochow, Jiangsu	A2063G	22	0	≥256	2–16	0.125–1	0.016–0.125
	None	0	2	< 0.008	< 0.008	0.25–0.5	0.032
Jinan, Shangdong	A2063G	8	3	128- > 256	2–32	0.125–1	0.032–0.125
	A2064G	1	0	256	2	0.5	0.032
	None	1	9	< 0.008	< 0.008	0.125–0.5	0.016–0.125
Fuyang, Anhui	A2063G	6	0	> 256	4–16	0.125–0.5	0.032–0.125
	None	0	2	< 0.008	< 0.008	0.25–0.5	0.032–0.125
Total	A2063G	114	7	128- > 256	2–32	0.125–1	0.016–0.25
	A2064G	2	0	≥256	2	0.5	0.032–0.125
	None	2	29	< 0.008	< 0.008	0.125–0.5	0.016–0.125

The MIC of each agent was defined as the lowest concentration of each antibiotic that prevented the color change

*ERY* erythromycin, *AZM* azithromycin, *LVX* levofloxacin, *TET* tetracycline

### Amplification and sequencing domains V of the 23S rRNA gene

A2063G mutation in domain V of the 23S rRNA gene was identified in 121 (98.3%) macrolide-resistant clinical isolates. Only two macrolide-resistant clinical isolates harbored the A2064G mutation in 23S rRNA gene. No domain V mutation was identified in 31 macrolide-susceptible clinical isolates. Furthermore, no other mutations were found in domain V of the 23S rRNA gene in all isolates (Table 3).

## Discussion

We analyzed the molecular biological features and antimicrobial susceptibility of *M. pneumoniae* isolates in 5 cities in China mainland between January 2017 and December 2018. As is known to all, *M. pneumoniae* is classified into genotype I and genotype II according to the genome and proteomics data [25–27]. To further distinguish the genome information, Degrange et al. established the MLVA genotyping technique based on 5 loci (i.e. Mpn1 and Mpn13–16), with which *M. pneumoniae* was divided into dozens of genotypes [28]. Afterwards, such technique has been extensively utilized in the scientific research. However, Mpn1 locus with the highest efficiency in classification is unstable [29]. In 2010, the Mpn1 locus was no longer proposed by the ESCMID Study Group for Mycoplasma Infections [23], but the MLVA genotyping capacity of Mpn13–16 showed significant decline. Based on 10 articles [16, 28–36] involving 1,122 *M. pneumoniae* isolates, the replication copy of Mpn13 and Mpn15 were highly consistent with genotype I and II. Among the 1,122 isolates, 1,074 (95.7%) with a replication copy of 4 and 7 for Mpn13 and Mpn15 loci showed genotype I, while those with a replication copy of 5 and 6 for Mpn13 and Mpn15 loci showed genotype II. In this study, the MLVA genotyping data were completely in line with these features, with more than 90% of the genotype I and II isolates showing a MLVA genotype of 4–5–7-2 and 3–5–6-2. This indicated that compared to the conventional genotyping technique, merely 7.8% (12/154) of the isolates with multiple genotypes could be divided using MLVA genotyping technique (Table 3). Recently, Xue et al. [20] investigated the MLVA genotypes of *M. pneumoniae* in Kunming City, which indicated that there was a base deletion (7 bp) in the repeats of Mpn15 in two patients. This imply that there are indeed some limitations in the current MLVA genotyping loci.

**Fig. 2:**
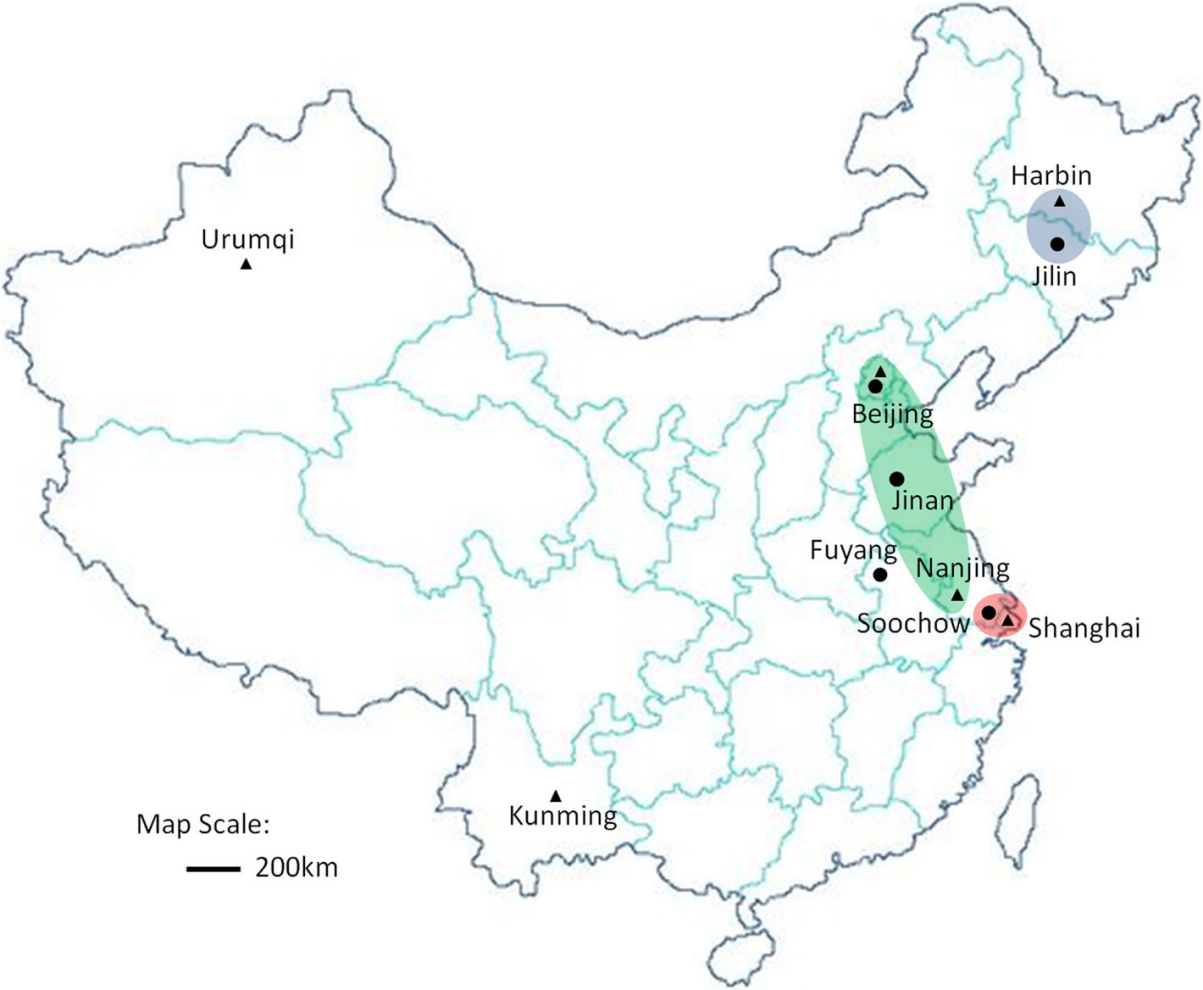
Locality map of ten cities included in this study(●) and newly publication(▲ Ref [21]). The genotyping and ML resistance were nearly the same in the cities in the cycle

In this study, the most common *M. pneumoniae* genotype was genotype I (4–5–7-2), while the rest was genotype II (3–5–6-2). There were significant differences in the genotyping among the five cities. The proportion of genotype I in Jilin city was 100%, while that in Jinan was merely 45.5%. According to the previous description [20], in geographic locations, Jilin and Harbin localizing in the Northeast of China with a linear distance of about 200 km showed a genotype I prevalence of 100 and 71.4%, respectively. In addition, the variations of genotypes in Nanjing and Shanghai with a linear distance of about 280 km were extremely large. Soochow, localizing at the middle of Nanjing and Shanghai in geography, showed a closer genotype I prevalence to Shanghai (91.7% vs. 80%). The most high prevalence of *M. pneumoniae* genotype II in the five cities was Nanjing, with a prevalence of up to 80%. In this study, the prevalence of genotype II in Beijing and Jinan, localizing at the north of Nanjing, was 54.5 and 33.3%, respectively **(**Fig. 2**)**. This implied that the distance may not be an important cause for variations in the *M. pneumoniae* genotypes.

The highest ML resistance proportion of isolates from Jilin, with the highest prevalence of genotype I, was 100%. Jinan, with the lowest prevalence of genotype I, showed the lowest ML resistance proportion of 54.5%. It seemed that ML resistance was correlated with the *M. pneumoniae* genotype (Tables 1 and 3, Fig. 1). Such phenomenon had been reported by other studies in China mainland [15, 17, 37, 38] rather than the other countries [24, 39, 40]. To date, the ML resistance of *M. pneumoniae* has been acknowledged to be associated with the mutations in 2617, 2063 and 2064 loci in 23 s rRNA [1, 41]. In this study, mutations on 2063 and 2064 loci were identified in the ML resistant strains, and no mutations were found in the ML susceptible strains. In a previous study, Okaziki et al. reported transition of ML susceptible to ML resistant *M. pneumoniae* strains in the presence of ML exposure. Meanwhile, there were point mutations at 2,063 and 2,064 of the 23 s rRNA gene, which resulted in stable phenotype-related antimicrobial resistance [42]. Therefore, the ML resistance of *M. pneumoniae* may be related to the antibiotics pressure to some extent, which was a gradual process. Nowadays, ML antibiotics have been commonly utilized in Chinese population, especially children. What’s more, abuse of ML antibiotics is also reported in some regions. In this study, the time span was short and was similar with the cross-sectional studies. Our data might not reflect the correlation between ML resistance and genotype of *M. pneumoniae*. However, our team performed 10-year cohort studies on the molecular characteristics and antimicrobial susceptibility of *M. pneumoniae* in Beijing. Between 2008 and 2012, the predominant genotype of *M. pneumoniae* was genotype I [15, 43]. In 2013, the proportion of genotype II *M. pneumoniae* showed a trend of increase [44]. Between 2014 and 2016, the proportion of genotype II *M. pneumoniae* was in a range of 30 to 40% [45]. In this study, the proportion of genotype II *M. pneumoniae* in Beijing between 2017 and 2018 was 39.3%. The prevalence of antimicrobially resistant genotype II *M. pneumoniae* in Beijing increased from 0 to 20% over the past decade [44, 45], which indicated that the prevalence of genotype II *M. pneumoniae* showed a gradual trend in the presence of antibiotics exposure. It seemed that the total antimicrobial resistance rates showed decline in Beijing [20, 45], but in fact, the antimicrobial resistance proportion of genotype I strains was high with no trend of decline, while the proportion of antimicrobial resistance in genotype II showed gradual increase. The decline of total antimicrobial resistance rates may be related to the rapid increase of genotype II *M. pneumoniae* in Beijing within these years compared to the increase of its antimicrobial resistance. In 2016, Xue et al. [20] reported the proportion of genotype II *M. pneumoniae* in Beijing was lower than the reported data (10.9% vs. 39.3%), which may be related to the variations induced by population and determination methods. In future, more persistent studies are required in order to obtain more accurate data.

Although the total ML resistance rate of *M. pneumoniae* among the 5 cities were in a range of 54.4 to 100%, the resistance rate of genotype I *M. pneumoniae* after genotyping was higher than 90% in all these regions (Table 4). The major causes for variations of the ML resistance were proportion and antimicrobial resistance rate of genotype II strains. There were only 2 genotype II strains isolated from Fuyang and Soochow, and then data analysis was not performed. The antimicrobial resistance rate of the genotype II strains (MLVA, 3–5–6-2 genotype) in Beijing and Jinan were all higher than 20%. This indicated that more studies are required to support the correlation between genotype II (MLVA, 3–5–6-2 genotype) and ML resistance. The proportion of genotype I and II strains in Jinan was similar. However, the rate of antimicrobial resistance of genotype I was 90%, while that of the genotype II was merely 25%. On this basis, we speculated that there might be a transmission between genotype I and II in Jinan, rather than the transmission from genotype II to genotype I. Our future studies will focus on it. Our study contributes to the understanding on the molecular epidemics of *M. pneumoniae* in China mainland, the drug resistance and clinical medication in different regions. In future, more studies involving a large sample size and more monitoring sites are required.

**Table 4:** Prevalence of macrolide resistance and genotyping in *M. pneumoniae* clinical isolates in five cities

Region	Total macrolide resistance rate	Percentage of Genotype II %	Macrolide resistance rate%	
			Genotype I	Genotype II
Jilin, Jilin	100	N	100 (49/49)	N (−)
Soochow, Jiangsu	91.6	8.3	100 (22/22)	N (0/2)
Fuyang, Anhui	75	25	100 (6/6)	N (0/2)
Beijing	66.7	39.3	96.8 (30/31)	20 (4/20)
Jinan, Shangdong	54.5	54.5	90 (9/10)	25 (3/12)

### Bias and limitations

There are some limitations in our study. Firstly, the sample size of strains in some regions was small. Secondly, the study duration was relatively short. It was similar to the cross-sectional study lacking of cohort studies. Thirdly, we only included five cities in this study, and our data could not represent the whole situation of *M. pneumoniae* infection in China mainland.

## Conclusions

In summary, we investigated the molecular characteristics of *M. pneumoniae* in 5 cities between January 2017 and December 2018. A large variance was identified in the *M. pneumoniae* genotype and ML resistance among the 5 cities. The proportion of *M. pneumoniae* with a genotype II (3–5–6-2) showed an increased trend, with an antimicrobial resistance of 0–25%. In future, more studies are required to confirm the correlation between specific *M. pneumoniae* genotype and ML resistance.

## Additional file

Additional file 1: All M. pneumoniae clinical isolates details from five cities. (XLSX 19 kb)

## Data Availability

The datasets used and/or analysed during the current study are available from the corresponding author on reasonable request.

## Abbreviations

AZM: azithromycin
CAP: community acquired pneumonia
ERY: erythromycin
LVX: levofloxacin
MICs: minimum inhibitory concentrations
ML: macrolide
MLVA: multilocus variable number tandem repeat analysis
TET: tetracycline
VNTR: variable-number tandem-repeat
